# Exploring Redox Modulation of Plant UDP-Glucose Pyrophosphorylase

**DOI:** 10.3390/ijms24108914

**Published:** 2023-05-17

**Authors:** Daniel Decker, Juliette Aubert, Malgorzata Wilczynska, Leszek A. Kleczkowski

**Affiliations:** 1 Department of Plant Physiology, Umeå Plant Science Centre, Umeå University, 90187 Umeå, Sweden; danielodecker86@gmail.com (D.D.); juliette.aub@gmail.com (J.A.); 2 Diamyd Medical, 90621 Umeå, Sweden; malgorzata.wilczynska@diamyd.com

**Keywords:** carbohydrate metabolism, glutathione, hydrogen peroxide, protein structure, redox regulation, substrate affinity, UDP-glucose pyrophosphorylase

## Abstract

UDP-glucose (UDPG) pyrophosphorylase (UGPase) catalyzes a reversible reaction, producing UDPG, which serves as an essential precursor for hundreds of glycosyltransferases in all organisms. In this study, activities of purified UGPases from sugarcane and barley were found to be reversibly redox modulated in vitro through oxidation by hydrogen peroxide or oxidized glutathione (GSSG) and through reduction by dithiothreitol or glutathione. Generally, while oxidative treatment decreased UGPase activity, a subsequent reduction restored the activity. The oxidized enzyme had increased *K*_m_ values with substrates, especially pyrophosphate. The increased *K*_m_ values were also observed, regardless of redox status, for UGPase cysteine mutants (Cys102Ser and Cys99Ser for sugarcane and barley UGPases, respectively). However, activities and substrate affinities (*K*_m_s) of sugarcane Cys102Ser mutant, but not barley Cys99Ser, were still prone to redox modulation. The data suggest that plant UGPase is subject to redox control primarily via changes in the redox status of a single cysteine. Other cysteines may also, to some extent, contribute to UGPase redox status, as seen for sugarcane enzymes. The results are discussed with respect to earlier reported details of redox modulation of eukaryotic UGPases and regarding the structure/function properties of these proteins.

## 1. Introduction

UDP-glucose (UDPG) pyrophosphorylase, a cytosolic protein, is a key enzyme of carbohydrate biosynthesis, producing UDPG, which serves as a substrate (or precursor) for the biosynthesis of a plethora of oligo- and polysaccharides, including sucrose (the main carbon transporting molecule in plants) as well as cellulose, hemicellulose, and other cell wall components [1,2,3,4,5]. Although UDPG production is thought to represent the major function of UGPase, its reaction is fully reversible. From thermodynamic principles, because of its reversibility and high activity, UGPase is considered an “equilibrating” enzyme coupled through UDPG production to several slow irreversible reactions (“engine” enzymes) [6,7,8]. UGPase uses UTP and glucose-1-P as substrates in the so-called forward direction of the reaction, as well as UDPG and pyrophosphate (PPi) as substrates in the reverse direction. As a consequence, UGPase sits at the crucial junction of plant metabolism, being indirectly involved in the synthesis of complex carbohydrates as well as in sugar and energy metabolism [8,9,10]. A central role in integrating primary metabolism was also proposed for UGPases from other eukaryotes [11,12,13].

Plant UGPase has been well characterized with respect to its physical/kinetic properties and regulation [5,14,15,16,17]. Surprisingly, however, no focused studies were carried out on possible redox modulation of the higher plant enzyme, a process known to affect hundreds of proteins [18,19]. Earlier studies on simple single-celled eukaryotes have revealed that UGPase activity is modulated by a redox mechanism involving oxidation and reduction of specific cysteine (Cys) residues [12,20,21]. For plant UGPases, the first hints of its possible redox control came from high-throughput proteomics studies [22,23], where seed UGPase was identified as one of many targets for in vivo interaction with thioredoxins, small proteins mediating redox control during oxidative stress conditions [24]. UGPase was also one of many protein targets that were S-glutathionylated at Cys residues in Arabidopsis cell culture during oxidative stress [25]. Glutathione (GSH), a peptide containing three amino acids (Glu-Cys-Gly), serves important functions in plants as a reductant and, along with its oxidized form (GSSG), is well known to participate in plant oxidative stress responses by reversibly modifying selected proteinaceous Cys residues [26,27,28,29]. More recently, a report by Soares et al. [30] demonstrated that the activity of purified sugarcane UGPase decreased upon oxidation by hydrogen peroxide (H_2_O_2_), whereas subsequent reduction by dithiothreitol (DTT) restored the activity. Since H_2_O_2_ is well known as a cellular signal of oxidative stress [31], whereas DTT can mimic the effects of thioredoxins and GSH [26,32,33], this suggested that redox modulation may indeed represent yet another level of regulation of plant UGPases in vivo.

Crystal structures of Arabidopsis and sugarcane UGPases have revealed that the proteins contain a Cys residue (Cys95 and Cys102, respectively) at the so-called nucleotide-binding (NB) loop [34,35]. Earlier kinetic studies on a barley UGPase mutant lacking Cys99 (homologous to Arabidopsis Cys95 and sugarcane Cys102) demonstrated that it had a decreased affinity for its substrates, especially for PPi [36].

To examine in more detail the redox control of plant UGPase activity, in the present study, we used purified wild-type (wt) UGPases from both sugarcane and barley. In addition, their respective mutants lacking a single Cys at the NB loop were used to check the role of this Cys in redox responses. Both sugarcane and barley wt UGPases were sensitive to redox modulation, which affected both the activity and substrate affinity of the enzymes, with a major, but not exclusive, involvement of the Cys99 and Cys102 of barley and sugarcane UGPases, respectively. We have also shown that glutathione in its reduced (GSH) and oxidized (GSSG) forms can readily substitute for DTT and H_2_O_2_, respectively, as effective modulators of UGPase activity. The results are discussed with respect to the structure-function properties of plant UGPase.

## 2. Results 

### 2.1. Redox Modulation of Sugarcane UGPase

**Figure 1:**
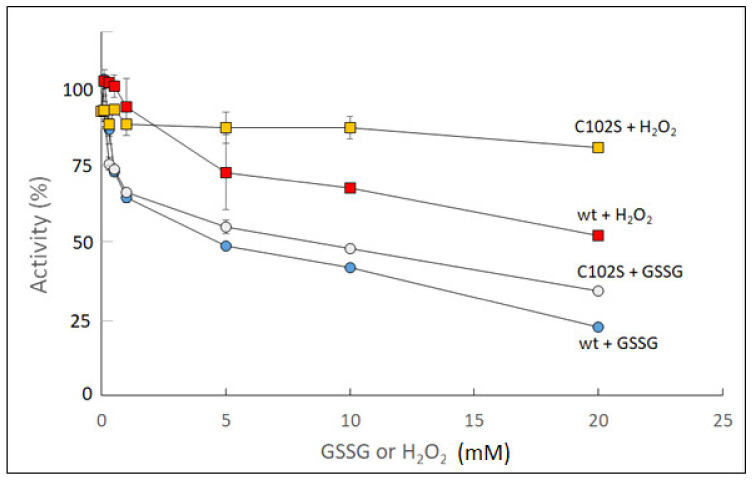
Effects of oxidized glutathione (GSSG) and H_2_O_2_ on activities of wt and Cys102Ser (C102S) mutant of sugarcane UGPase. The proteins were incubated for 30 min (at RT) at a given concentration of the oxidant or in water (no oxidant).

To study redox effects on sugarcane UGPase, we used both wt protein and its Cys102Ser mutant and treated them with either H_2_O_2_ or oxidized glutathione (GSSG). Oxidation by up to 20 mM of GSSG or H_2_O_2_ for 30 min led to a decrease in activity for both wt and Cys102Ser mutant enzymes, and the effects of GSSG were stronger than those of H_2_O_2_. The mutant appeared less sensitive to H_2_O_2_ treatment but was almost as strongly affected as wt by GSSG (Figure 1). Both H_2_O_2_ and GSSG had stronger inhibitory effects on sugarcane wt UGPase activity when compared to its Cys102Ser mutant (Figure 1).

**Figure 2:**
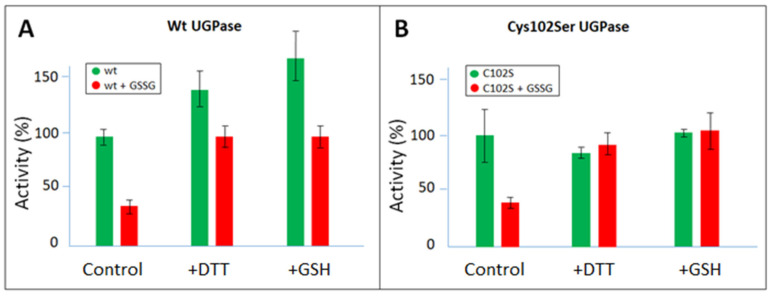
Effects of dithiothreitol (DTT) and glutathione (GSH) on S-glutathionylated wt sugarcane UGPase (**A**) and its Cys102Ser (C102S) mutant (**B**). The proteins were incubated at RT for 30 min ± 10 mM GSSG (control). This was followed by treatment with 20 mM DTT or 20 mM GSH for 3 h.

The GSSG-treated enzyme could regain its activity when reduced by DTT or GSH, and this concerned both wt UGPase and its Cys102Ser mutant (Figure 2), indicating that the oxidation process could be reversed by the reductant. The GSSG-oxidized sugarcane proteins were more sensitive to DTT and GSH reduction than the untreated enzymes (Figure 2). Earlier, the DTT-stimulated recovery of activity was observed for H_2_O_2_-pre-treated sugarcane UGPase, but the effects of GSSG and GSH were not tested [30].

**Figure 3:**
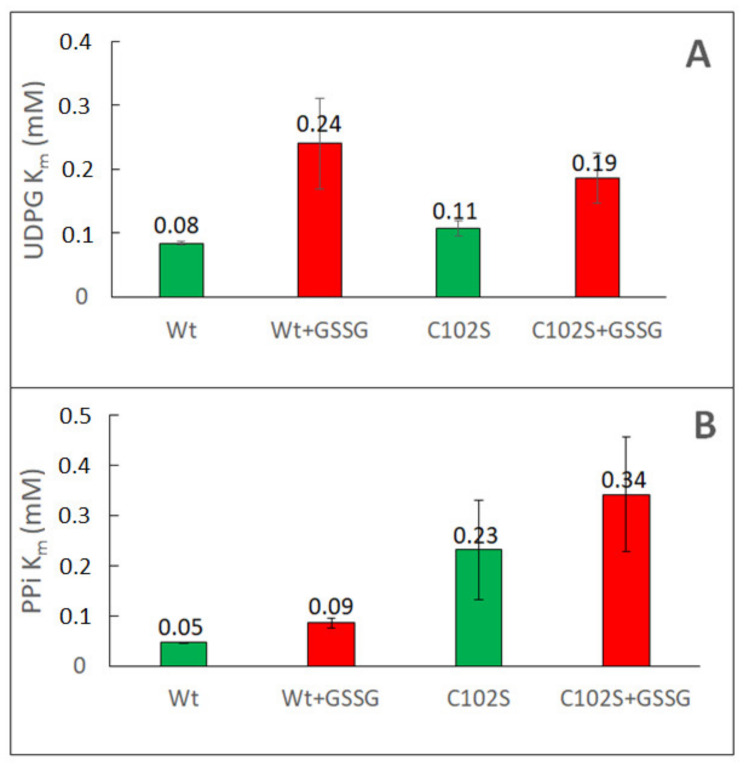
Comparison of *K*_m_ values for untreated and oxidized sugarcane wt UGPase and its Cys102Ser mutant (C102S). Panels (**A**,**B**) refer to *K*_m_ values with UDPG and with PPi, respectively. The *K*_m_s were obtained by the double reciprocal plot method (see Section 4 for details). Prior to assays, the proteins were incubated overnight with either water (“untreated” enzyme, green boxes) or 10 mM GSSG (“oxidized” enzyme”, red boxes). The determined *K*_m_ values are indicated on top of each box.

For kinetic characterization of sugarcane wt UGPase and its Cys102Ser mutant, we have determined *K*_m_ values for UDPG and PPi by varying the concentration of one substrate at the constant fixed level of the other substrate. The analyses were done both for the non-treated and GSSG-treated proteins (Figure 3). For either wt and the mutant, their *K*_m_ values with UDPG were roughly similar (0.08 and 0.11 mM, respectively), and both proteins displayed a decreased affinity for UDPG (increased *K_m_*) after oxidation by GSSG (Figure 3). The *K*_m_ with PP_i_, however, was much lower for wt UGPase than for the Cys102Ser mutant (0.05 mM versus 0.23 mM). Similar results were obtained earlier for purified barley UGPase and its Cys99Ser mutant, where the *K*_m_ with PPi of wt protein was 7–10-fold lower than that of the mutant [36,37].

Oxidation of sugarcane wt UGPase by GSSG led to a two–three-fold increase in its *K*_m_ values with UDPG and PPi, while the *K*_m_s of Cys102Ser protein also increased but were less strongly affected by GSSG (Figure 3). Nevertheless, in the case of the mutant, the GSSG-induced increase of its *K*_m_ values suggests that oxidation of Cys residues other than Cys102 may have also contributed to the lower substrate affinity of the oxidized enzyme (see Section 3).

### 2.2. Redox Modulation of Barley UGPase

Barley UGPase has been the primary model to study plant UGPases, both at the protein structure and enzyme regulation levels [5,36,37,38]. Thus, given the evidence on redox control of sugarcane UGPase (Figure 1, Figure 2 and Figure 3), it was of interest whether barley UGPase is also responsive to redox control. 

**Figure 4:**
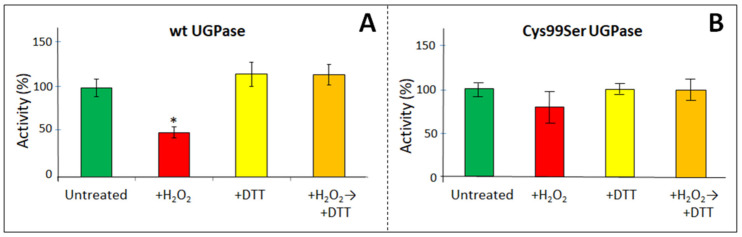
Redox modulation of wt and Cys99Ser mutant of barley UGPase. The proteins were incubated at RT for 10 h either untreated or with 0.5 mM H_2_O_2_ (for wt) or 5 mM H_2_O_2_ (for Cys99Ser mutant), followed by incubation with 10 mM DTT for 40 min (at RT). Assays contained 0.85 mM UDPG and 0.5 mM PPi. Panels (**A**,**B**) refer to wt UGPase and the Cys99Ser mutant, respectively. The asterix (*) refers to statistical significance between the samples (*p* < 0.05).

Compared to sugarcane wt UGPase, the barley wt enzyme was more sensitive to H_2_O_2_ treatment. Its activity was at least two-fold inhibited by oxidation by as little as 0.5 mM H_2_O_2_ (Figure 4A). This needs to be compared with the activity of sugarcane wt UGPase, which was inhibited by less than 50% only by 20 mM H_2_O_2_ (Figure 1). On the other hand, the Cys99Ser mutant was resistant to H_2_O_2_ treatment, showing only about 15% inhibition at 5 mM H_2_O_2_ (Figure 4B). The H_2_O_2_-oxidized wt and mutant proteins could regain all their activity after treatment with DTT (Figure 4B). The restoration of activity by DTT has indicated the reversibility of redox modulation of barley UGPase, similar to the sugarcane enzyme that was oxidized by GSSG and reduced by DTT or GSH (Figure 2).

**Figure 5:**
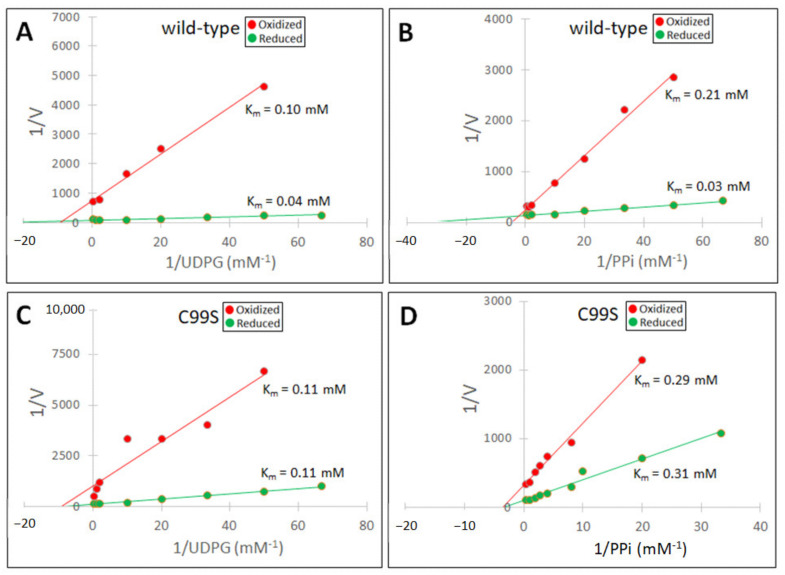
Determination of *K*_m_ values for barley wt UGPase and its Cys99Ser mutant (C99S) with UDPG (panels **A**,**C**) and PPi (panels **B**,**D**). Double reciprocal plots are shown, where UGPase activity (V) was measured at fixed concentrations of either PPi or UDPG, with the other substrate concentration varied. Prior to assays, the enzymes were incubated overnight at RT with either 2 mM H_2_O_2_ (“oxidized” enzyme) or 2 mM DTT (“reduced” enzyme). See Section 4 for other details.

The *K*_m_ values for barley UGPase were determined for purified wt and Cys99Ser proteins which were pre-incubated with either H_2_O_2_ or DTT (Figure 5). These conditions differed from those for *K*_m_ determinations of sugarcane UGPase, where purified wt and Cys102Ser proteins were pre-incubated with or without GSSG (Figure 3). However, in both cases, oxidation by either H_2_O_2_ (barley enzyme) or GSSG (sugarcane enzyme) resulted in similar effects on *K*_m_ values. Thus, the oxidation led to a ca. two-fold increase in the *K*_m_ with UDPG for both wt and mutant enzymes and a several-fold increase in the *K*_m_ with PPi for wt UGPases, but not for the mutants (Figure 3 and Figure 5). Treatment with H_2_O_2_ or DTT had no effect on the *K*_m_ values of the barley Cys99Ser mutant, and those values corresponded to the *K*_m_s of oxidized wt protein (Figure 5). This suggests that Cys99, and no other Cys residues, is essential for redox modulation of barley UGPase. Supporting this is little or no effect of 5 mM H_2_O_2_ on the activity of the mutant (Figure 4B).

## 3. Discussion

### 3.1. Redox Compounds Affecting Plant UGPase Activity

Generally, we found that plant UGPase is sensitive to oxidation by H_2_O_2_ and GSSG (e.g., Figure 1). These two compounds are believed to be the main players during oxidative stress conditions, and they regulate the activities of hundreds of proteins via post-translational modification of selected Cys residues [18]. The oxidation resulted in lower activities for both barley and sugarcane UGPases (Figure 1, Figure 2 and Figure 4). This decrease in activity could be reverted by the treatment of oxidized UGPase with DTT or GSH (Figure 2 and Figure 4).

Both H_2_O_2_ and GSSG cause oxidation of proteinaceous Cys residues, but their modes of action are different. Whereas H_2_O_2_ oxidizes the -SH group of a Cys to sulfenic acid (-SOH) [38], the effect of GSSG is to oxidize (glutathionylate) the -SH group to a bulky -SSG [27]. In most organisms and in cell culture, the concentration of H_2_O_2_ was estimated at extremely low levels, from pico- to nanomolar [39,40]. At low levels, it acts as a signaling molecule, and at high levels, it induces cell death [41]. There are exceptions, however, e.g., in sugarcane leaves but not sugarcane stem internodes, H_2_O_2_ was reported at up to 50 mM [30]. Glutathione, on the other hand, is present at millimolar concentrations in all aerobic organisms, and the GSSG/GSH ratio is an important indicator of oxidative stress conditions [42].

UGPase is one of many proteins that undergo S-glutathionylation, as found for Arabidopsis [25]. The GSSG (along with its reduced form, GSH) is believed to be involved in redox regulation in vivo of a variety of proteins, especially those located in the cytosol, in both plant and animal cells [43,44]. In most cases, the process involves the use of GSSG for a reversible modification of protein cysteinyl residues that can directly modulate a given protein activity [29]. In barley, using a genetically-encoded biosensor of cytosolic glutathione redox potential, this potential was found to be highly robust under combined salt and osmotic stresses [45]. This is consistent with plant UGPase activity being responsive to these stresses [46,47,48].

### 3.2. Redox and Substrate Affinity of Plant UGPases

In our earlier work on barley UGPase mutants, the Cys99Ser mutant had characteristically increased *K*_m_ values (i.e., lower substrate affinity) with UDPG and, especially, with PPi, when compared to *K*_m_s of the wt enzyme [36,37]. Similar results were obtained in the present study for both barley Cys99Ser and sugarcane Cys102Ser mutants. As seen in Table 1, which lists *K*_m_ values for redox-modulated sugarcane and barley UGPases (based on Figure 3 and Figure 5), the untreated or reduced mutants had increased *K*_m_ values for both UDPG and PPi, when compared to their respective wt proteins. This was especially evident for *K*_m_ values with PPi, which were ca. six- and 10-fold higher for Cys102Ser and Cys99Ser mutants when compared to those of their wt counterparts. Upon oxidation of wt enzymes, their *K*_m_s with UDPG and PPi notably increased, especially in the case of *K*_m_ with PPi for barley wt UGPase (Table 1). On the other hand, oxidation of the mutants led only to a moderate increase in *K*_m_ values for the sugarcane Cys102Ser protein but had no effect on the substrate affinity of the Cys99Ser barley mutant (Table 1).

For both sugarcane and barley wt UGPases, the differences in *K_m_* values between non-treated/reduced and oxidized enzymes suggest that redox conditions affect their substrate affinity. For the sugarcane enzyme, the redox status of its Cys102 appears to be the major, but not the only, factor involved in these effects. In contrast, for barley UGPase, its Cys99 appears to be responsible for most, if not all, of redox effects. 

Redox-induced changes in substrate affinity (Table 1) may have important consequences for in vivo activities of plant UGPase, especially with respect to its affinity for PPi. Furthermore, PPi serves as a UGPase substrate only when complexed with Mg^2+^ (as MgPPi), whereas free PPi is a strong inhibitor of the enzyme [37,49]. Thus, under oxidative stress, the lower affinity of UGPase for PPi may lead to an increase in the cytosolic concentration of free PPi, resulting in a further decrease in the activity of this enzyme. 

### 3.3. Structural Basis for Redox Control of Plant UGPases

**Figure 6:**
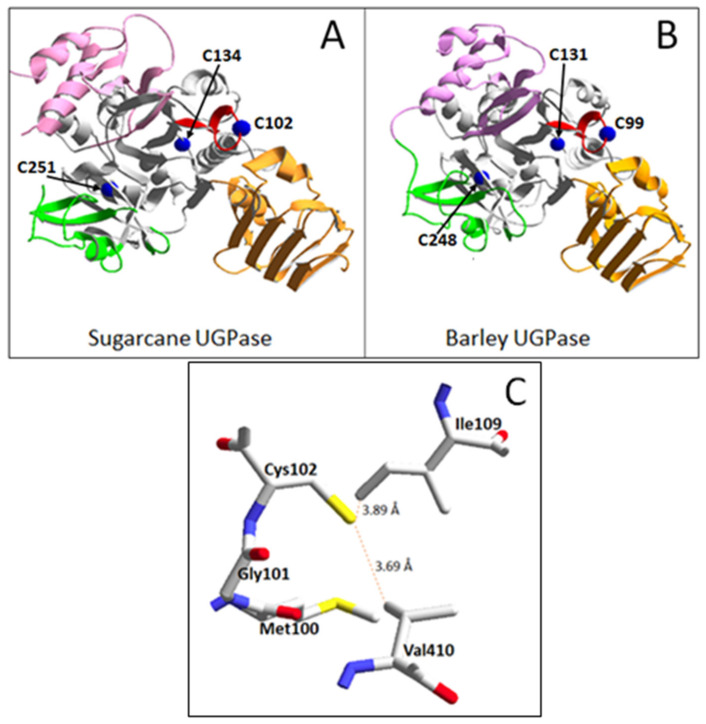
Structures of sugarcane and barley UGPases. Structures shown are based on the crystal structure of sugarcane UGPase (pdb 5WEG) [35] and for homology-modeled barley UGPase, based on Arabidopsis UGPase crystal structure (pdb 1z90) [37]. (**A**) General view of sugarcane UGPase, with the N-terminal domain on the upper left (pink color), central domain (gray), and C-terminal domain on the lower right (orange). Shown also is the sugar-binding domain (green) and nucleotide-binding (NB) loop (red). The position and numbering of three cysteine molecules are indicated by blue balls corresponding to their α-carbons. (**B**) A similar view of barley UGPase, with its three Cys numbered and shown as blue balls. UGPase active site is composed of specific amino acids from the N-terminal domain, sugar-binding domain, central domain, NB loop, and C-terminal domain [34]. (**C**) The environment of Cys102 in sugarcane UGPase (pdb 5WEG) [35]. Shown are the closest amino acids at a distance of 4 Å from the sulfur atom of Cys 102. Broken red lines refer to distances from Cys102 to Ile109 (located on NB loop, central domain) and Val410 (C-terminal domain), which are likely to interplay with the Cys via hydrophobic bonds.

Amino acid sequences of both sugarcane and barley UGPases contain only three Cys residues: Cys102, Cys134, and Cys251 for sugarcane UGPase; and Cys99, Cys131, and Cys248 for the barley protein. Sugarcane UGPase Cys102 and its barley homolog Cys99 are located at the NB loop that is close to the active site of the protein [34,35,37], whereas the other two Cys are more distant and are located in the central domain (Figure 6A,B). For the three Cys residues, only Cys102 (sugarcane protein) and Cys99 (barley protein) are conserved in all eukaryotic UGPases, whereas other Cys are conserved for UGPases only in plants [12,20,30]. If the three Cys molecules are analyzed in pairs, their relative distances range between 23 and 35 Å from each other; this is too far for any pair of these Cys to form a disulfide bridge (S-S). In comparison, a typical distance between Cys residues in a protein structure to form an S-S bond is 2.03 Å [12]. Thus, it seems unlikely that the redox effect on plant UGPase involves disulfide bond formation for its internal Cys residues. More likely, the UGPase Cys residues are reversibly S-glutathionylated and S-sulfenylated by GSSG and H_2_O_2_, respectively. The -SH group of Cys that was oxidized by either H_2_O_2_ or GSSG can be reduced back by either GSH, DTT, or reduced thioredoxin [50]. Seed UGPases from wheat and *Medicago truncatula* have already been identified as in vivo targets for thioredoxin reduction [22,23].

Among eukaryotic UGPases, only the enzyme from *Entamoeba histolytica*, a single-celled parasite, was demonstrated to be redox modulated via reversible disulfide bond formation for two of its Cys residues [12], and this was shown by detailed modeling and SDS-PAGE analyses of oxidized and reduced UGPase protein. The Cys molecules involved were Cys108 and Cys378 [12], the former corresponding to Cys99 and Cys102 of barley and sugarcane UGPases, respectively [30,36].

For sugarcane UGPase, two of the near neighbors of Cys102 are Ile109 (also located on the NB loop) and Val410 (from the C-terminal domain) (Figure 6C). Corresponding configurations are also present in Arabidopsis and barley UGPases. As hydrophobic interactions usually have carbon-carbon distances of 3.3–4.0 Å, the short distances shown in Figure 6C suggest that Cys 102 is involved in hydrophobic interplay with these amino acids. Cysteine, despite possessing a polar sulfhydryl group, frequently tends to behave as a hydrophobic (rather than polar) residue in folded protein structures [51].

It is unknown how the oxidation of a specific Cys residue (Cys102 and Cys99 for sugarcane and barley UGPases, respectively) inhibits UGPase activity and leads to an increase of *K*_m_ values for its substrates. This Cys, located near the tip of the NB loop (Figure 6), is distant from the active site of each enzyme. However, the loop contains three other amino acids known to coordinate substrate binding. In Arabidopsis UGPase, these amino acids are Leu85—coordinating ribose moiety of UTP; Gly87—uridyl group; and Lys99—phosphate moiety (all numbered as in Arabidopsis UGPase) [34]. These amino acids have their homologs in sugarcane and barley UGPases. Thus, it is tempting to suggest that oxidation of the NB-loop-located Cys may affect the conformation of the NB loop itself, which in turn could affect the binding of substrates in the active site. A similar explanation may also be given for low substrate affinity (higher *K*_m_ values) of the Cys99Ser and Cys102Ser mutants used in this study. Taking into account the hydrophobic environment for the NB-located Cys (Figure 6C), replacing it with Ser (a hydrophilic amino acid) could not only affect the conformation of the NB loop but also disrupt inter-domain hydrophobic interactions (through Val410). More detailed studies are required to address this.

It also remains to be investigated whether redox modulation of UGPase affects its tertiary structure. Plant UGPases are active as monomers only, but under some conditions, they can form inactive dimers and higher-order oligomers [30,34,35,36,37,52,53,54]. In comparison, human UGPase is active only as an octamer, and octamer formation is essential for its activity [13,55], even though the human and plant enzymes share over 50% identity at their amino acid sequences [38]. In sugarcane, UGPase was present as both a monomer and a dimer in the leaves but only as a monomer in the stem internodes [30]. In the same study, sugarcane leaves, but not internodes, were reported to accumulate large amounts (up to 50 mM) of H_2_O_2_. This inferred that H_2_O_2_ might affect the quaternary structure of UGPase, and, thus, its activity [30]. Earlier investigations of UGPase from *Euglena gracilis*, a simple single-cell photosynthetic organism, have suggested that, after oxidation by H_2_O_2_, the enzyme arranges in several enzymatically-inactive structural conformations, both in vitro and in vivo [21]. The reported presence of UGPase monomers and dimers in sugarcane leaves containing high [H_2_O_2_] [30] suggests that oligomerization is a regulatory mechanism controlling UGPase activity in response to oxidative stress; however, its exact mechanism is still unknown.

### 3.4. Plant UGPase Is under Transcriptional, Posttranslational, and Metabolite Control

Over the years, it has become clear that UGPase is regulated at several levels, including transcriptional (at gene expression level), post-translational (at protein level), and metabolic regulation (at activity level) [5]. Each of these modes of regulation on their own may considerably affect UGPase activity and, subsequently, the flow of carbon in overall carbohydrate metabolism in both photosynthetic and non-photosynthetic tissues. Those modes of control are frequently overlapping, thus making UGPase activity even more sensitive to their regulation.

Concerning transcriptional control, UGPase gene expression was already shown as strongly affected by light-dark transition, low temperature, phosphate deficiency, salt stress, heavy metal stress, and exposure to sucrose and other sugars [30,46,53,56,57,58,59,60]. In many cases, the amount of UGPase protein and its activity were roughly correlated with changes in UGPase gene expression. As common in such studies, it is difficult to differentiate whether changes in UGPase activity during such stresses are brought about by direct effects of the stressor on UGPase (e.g., affecting the stability of the protein) or indirectly via effects on UGPase gene expression or both [16]. A good example is H_2_O_2_, which besides directly affecting UGPase activity and its substrate affinity, as shown in this study (Figure 3, Figure 4 and Figure 5), is known to affect the expression of a multitude of genes [61]. At low concentrations, H_2_O_2_ acts as a messenger molecule triggering tolerance against various abiotic stresses [31], while at high concentrations, it orchestrates programmed cell death [62].

Plant UGPases have been shown to be post-translationally modified in a number of ways. Examples include phosphorylation of Ser419 of sugarcane UGPase [30], binding to 14-3-3 proteins for the enzymes from barley and Arabidopsis [63,64], rice UGPase acetylation [65], and N-glycosylation of rice and maize UGPases [66,67]. It appears now that also redox modulation, as shown here for sugarcane and barley UGPases, needs to be added to this ever-growing list.

Substrate availability and product inhibition may also affect UGPase activity [15,68]. UGPase, by carrying a fully reversible reaction, may be involved in anabolic pathways by using UTP and glucose-1-P to produce PPi and UDPG (the latter serving as a key precursor of oligo- and polysaccharides), but also—in its reverse reaction—in catabolic processes, where glucose-1-P is broken down during respiration [5,8,49]. Thus, the direction of the UGPase reaction depends on the overall metabolic status of a tissue and may change depending on environmental/stress factors. Strong product inhibition by PPi of the forward UGPase reaction is a well-known metabolic mechanism [15,49], modulating the direction of carbohydrate metabolism in vivo [68]. A number of chemical inhibitors have been described that strongly inhibit UGPase activity (at a µM range) both in vitro and in planta [69,70]; however, all of them also affect the activities of so-called UDP-sugar pyrophosphorylases, which are related to UGPase in that they can non-specifically produce UDPG or use it as a substrate [71].

Finally, the UGPase reaction and its reversibility depend very much on the concentration of free magnesium (Mg^2+^), which regulates the formation of both MgATP and MgPPi complexes [72] that are true substrates/products of UGPase [49,73]. Changes in [Mg^2+^] reflect the energy status of a given tissue and arise mostly from intracellular pools of adenylates, uridylates, and other nucleotides [74,75,76]. Adequate intracellular [Mg^2+^] has already been shown to ease oxidative stress by affecting the phloem transport of sugars and controlling optimal CO_2_ fixation, especially under high-light conditions [77].

More detailed studies are required on the redox status of the NB-located Cys and other Cys residues of plant UGPase, involving MALDI-TOF mass spectrometry analyses. This should also include an investigation of the redox status of methionine (Met) residues. Met can have the same function as Cys residues when it comes to redox regulation [78]. There is also a possibility that plant UGPases contain a non-catalytic binding site(s) for MgPPi, as reported, e.g., for *E. coli* ATPase [79]. Overall, this would give a more profound picture of redox regulation at the UGPase active site and potentially on alternative binding sites.

In conclusion, redox modulation of plant UGPase, as shown here for the enzymes from sugarcane and barley, adds yet another mode of regulation to a long list of mechanisms affecting UGPase activity. There is a variety of abiotic factors that give rise to oxidative stress conditions, e.g., drought, high temperature, high light intensity, heavy metals, salinity, or ultraviolet radiation, and they all cause yield and quality losses in crops. Some of these abiotic factors are transient, as they are part of normal weather conditions, but some may persist and overlap during a plant life span [80]. With UGPase positioned at the very center of primary plant metabolism, its redox regulation may actually turn out to be a major mechanism controlling plant yield.

## 4. Materials and Methods

### 4.1. Materials

Constructs containing wt and Cys102Ser mutant of sugarcane UGPase were prepared as in [30,35] and were kindly provided by Dr. José Sérgio Soares, Functional Genome Lab., State Univ. Campinas, Brazil. Recombinant sugarcane UGPases were purified by the Umeå University Protein Expertise Platform (PEP) by immobilized metal (nickel) affinity chromatography, and the polyHis tail was cleaved off by TEV protease. Aliquots of purified enzyme, in concentrations of 0.3–0.45 mg/mL, were stored at −80 °C in 29 mM phosphate buffer (pH 8.0), 150 mM NaCl and 1 mM β-mercaptoethanol. Purified recombinant barley UGPase and its Cys99Ser mutant were produced as described in [36,37].

### 4.2. Assays

The recombinant sugarcane UGPase was assayed at room temperature (RT) in the reaction mixture containing 100 mM Hepes-NaOH (pH 7.5), 5 mM MgCl_2_, 0.6 mM NADP, 1 unit each of coupling enzymes: phosphoglucomutase and glucose 6-phosphate dehydrogenase. Unless otherwise stated, PP_i_ and UDPG (substrates used) were kept at 1 mM and 2 mM, respectively. The reactions, at a final volume of 100 µL, were started with an aliquot of UGPase. Assays were performed in 96 well plates (Sarstedt, Numbrecht, Germany), and the formation of NADPH was determined at 340 nm on a Spectramax 190 plate reader (Molecular Devices, Sunnyvale, CA, USA). The assays of recombinant barley UGPase were carried out essentially as those for sugarcane enzyme, with the exception that, unless otherwise stated, UDPG and PPi were at 1 mM each.

One unit of UGPase activity refers to 1 µmol NADPH formed per minute under assay conditions, corresponding to 1 µmol of glucose-1-P (product of UGPase) formed during the reaction. Specific activities of sugarcane UGPase, as found in this study, were 480 unit/mg and 390 unit/mg for wt and Cys102Ser proteins, respectively; for barley UGPase, they were 1200 unit/mg for wt and 650 unit/mg protein for Cys99Ser mutant, respectively [36]. Assay chemicals were obtained from Sigma-Aldrich Sweden AB, Stockholm, Sweden.

### 4.3. Kinetic Studies

For the determination of Michaelis–Menten constant (*K*_m_) values for UDPG and PPi, assays were done by varying the concentration of one substrate at the constant fixed level of the other substrate. For *K*_m_ determinations of sugarcane UGPase, fixed concentrations of substrates were: 2 mM UDPG and 1 mM PPi. For barley UGPase, both UDPG and PPi were fixed at 1 mM (for wt) and at 2 and 5 mM, respectively, for the Cys99Ser mutant. The *K*_m_ values were calculated using the Excel solver add-in. Kinetic points were means of at least two repeats that were reproducible within ±10%. Prior to assays, sugarcane UGPase was pre-incubated overnight at RT either untreated or with 10 mM GSSG (oxidized enzyme). Before assays of barley UGPase, the enzyme was preincubated overnight at RT with 2 mM DTT (reduced enzyme) or 2 mM H_2_O_2_ (oxidized enzyme).

### 4.4. Structural Modeling

Barley UGPase 3D structure was homology-modeled, based on the crystal structure of Arabidopsis UGPase1 [34], as in [37,54]. Both the barley UGPase homology model and the crystal structure of sugarcane UGPase (pdb 5WEG) [35] were displayed by DeepView/Swiss-PdbViewer (v4.1) (https://spdbv.unil.ch/) [81].

## Figures and Tables

**Table 1:** Summary of the redox effects on *K*_m_ values of UGPases from sugarcane (wt and Cys102Ser) and barley (wt and Cys99Ser mutant). Compiled from Figure 3 and Figure 5.

**Sugarcane UGPase**	**Wild type**			**Cys102Ser**	
	Untreated	Oxidized		Untreated	Oxidized
*K_m_* UDPG (mM)	0.08	0.24		0.11	0.19
*K_m_* PPi (mM)	0.04	0.09		0.23	0.34
					
**Barley UGPase**	**Wild type**			**Cys99Ser**	
	Reduced	Oxidized		Reduced	Oxidized
*K_m_* UDPG (mM)	0.04	0.10		0.11	0.11
*K_m_* PPi (mM)	0.03	0.21		0.31	0.29

## Data Availability

Data is contained within the article.
